# Using the Implementation Research Logic Model to design and implement community-based management of possible serious bacterial infection during COVID-19 pandemic in Ethiopia

**DOI:** 10.1186/s12913-022-08945-9

**Published:** 2022-12-13

**Authors:** Gizachew Tadele Tiruneh, Tsinuel Girma Nigatu, Hema Magge, Lisa Ruth Hirschhorn

**Affiliations:** 1 The Last Ten Kilometers (L10K) Project, JSI Research & Training Institute, Inc, Addis Ababa, Ethiopia; 2 Department of Pediatrics and Child Health, Jimma University, Ethiopia and Fenot Project - School of Population and Public Health, University of British Columbia, Vancouver, Canada; 3 Bill & Melinda Gates Foundation, Seattle, WA USA; 4 Feinberg School of Medicine and Havey Institute of Global Health, Northwestern University, Chicago, IL USA

**Keywords:** COVID-19, Ethiopia, Implementation science, Implementation research logic model, Implementation challenges, Implementation strategies, Neonatal sepsis, Newborn, Possible serious bacterial infection, Young infants

## Abstract

**Background:**

Community-based treatment of possible serious bacterial infection (PSBI) in young infants, when referral to a hospital is not possible, can result in high treatment coverage and low case fatality. However, in Ethiopia, the coverage of PSBI treatment remains low, worsened by COVID-19. To understand the challenges of delivery of PSBI treatment and design and test adaptative strategies to mitigate the impact of COVID-19 on neonatal mortality, we did implementation research (IR) employing Implementation Research Logic Model (IRLM). In this paper, we describe IRLM application experiences in designing, implementing, and evaluating strategies to improve community-based treatment of PSBI during the COVID-19 pandemic in Ethiopia.

**Methods:**

This IR was conducted between November 2020-April 2022 at Dembecha and Lume woredas of Amhara and Oromia regions, respectively. We employed narrative reviews, formative assessment and facilitated stakeholder engagement to develop the PSBI treatment IRLM to identify barriers, understand the conceptual linkages among determinants, choose implementation strategies, elicit mechanisms, and link to implementation outcomes. In addition, we used the IRLM to structure the capture of emerging implementation challenges and resulting strategy adaptations throughout implementation.

**Results:**

This IR identified COVID-19 and multiple pre-existing contextual factors. We designed and implemented implementation strategies to address these challenges. These adaptive strategies were implemented with sufficient strength to maintain the delivery of PSBI services and improve mothers’ care-seeking behavior for their sick young infants.

The IRLM offers us a clear process and path to prioritize implementation challenges, choose strategies informed by mechanisms of action, and where the adaptive implementation of community-based management of PSBI would lead to high-implementation fidelity and change in mother behavior to seek care for their sick young infants. The IRLM was also an effective tool for stakeholder engagement, easily explained and used to structure discussion and decision-making during co-design meetings.

**Conclusions:**

The use of the IRLM helps us to specify the conceptual links between the implementation challenges, strategies, mechanisms of action, and outcomes to explore the complex community-based management of PSBI during complex contexts to improve high-fidelity implementation and integration of PSBI treatment in the primary healthcare delivery systems through active engagement of stakeholders.

**Supplementary Information:**

The online version contains supplementary material available at 10.1186/s12913-022-08945-9.

## Background

Possible serious bacterial infection (PSBI) was identified as a leading cause of mortality in young infants (0–59 days of age) in Sub-Saharan Africa, contributing to 37% of the 2.1 million neonatal deaths [1]. Population-based surveillance data of young infants found a prevalence of 13.1% of one or more signs of infection [2], with an incidence of sepsis estimated to be 6.2% and a case fatality rate of 14.1% [3] in sub-Saharan Africa (as high up to 24% in low and middle-income countries [4–6]). The World Health Organization (WHO) recommends a simplified regimen (injectable and oral antibiotics) for community management of PSBI when referral to a hospital is not possible [7]. Evidence from Ethiopia, Malawi, Nigeria, Pakistan, and India [8–16] revealed that the simplified regimen scale-up for PSBI treatment results in high coverage and low case fatality.

Ethiopia introduced community-based newborn care (CBNC) in its flagship Health Extension Program (HEP) to improve access to and use of appropriate treatment for neonatal sepsis when a referral was not possible in 2012 [17]. However, the coverage of sick newborn care is low due to multiple factors: an overall decline in the performance of the HEP observed in recent years [18], low Health Extension Workers’ (HEWs) competency and motivation [9, 19, 20], erratic supply of essential drugs [19, 20], suboptimal supportive and referral links [9, 19, 20], communities’ misperceptions about newborn illnesses [21, 22], and socio-cultural beliefs and mothers’ limited decision-making power [19, 21–23]. In addition, there is a concern from program managers that the Coronavirus (COVID-19) pandemic has stressed the already weak healthcare system in Ethiopia, exacerbating the already fragile newborn and child health services.

There is limited evidence of the level of impact of the COVID-19 pandemic on care-seeking for neonatal sepsis programs in Ethiopia. Implementation research (IR) was conducted between November 2020-April 2022 in Dembecha and Lume districts of Amhara and Oromia regions, respectively. The research was carried out by JSI Research & Training Institute Inc./The Last Ten Kilometers (L10K) Project, funded by the Bill & Melinda Gates Foundation. The aim was to understand the demand- and supply-side challenges of delivery of PSBI treatment during the COVID-19 pandemic and to develop and test adaptative strategies to mitigate the impact of COVID-19 and other barriers on community-based management of PSBI implementation and uptake. Implementation research is the scientific study of methods to promote the systematic uptake of research findings and other evidence-based practices into routine practice, to improve the adoption, high-fidelity implementation, and maintenance of quality, effective, evidence-based practices in health care services [24]. The Implementation Research Logic Model (IRLM) combines a number of commonly used IR frameworks to help design or evaluate the implementation of evidence-based interventions by identifying determinants and selection strategies through mapping mechanisms and linking those implementation and effectiveness outcomes [25]. There is also emerging evidence from the United States that IRLM is an effective tool for stakeholder engagement critical for success in implementation work [26], but not in Ethiopia or similar settings.

In this paper, we describe the use of the IRLM for stakeholder engagement and incorporate formative research to inform the design, implementation, and evaluation of strategies to improve community-based management of neonatal bacterial infection during the COVID-19 pandemic. The utilization of the IRLM in Ethiopia represents important insights into using this tool and IR to inform adaptive implementation, engaging key stakeholders, and generating generalizable knowledge to inform the broader, context-adapted implementation of PSBI treatment.

## Settings

**Fig. 1:**
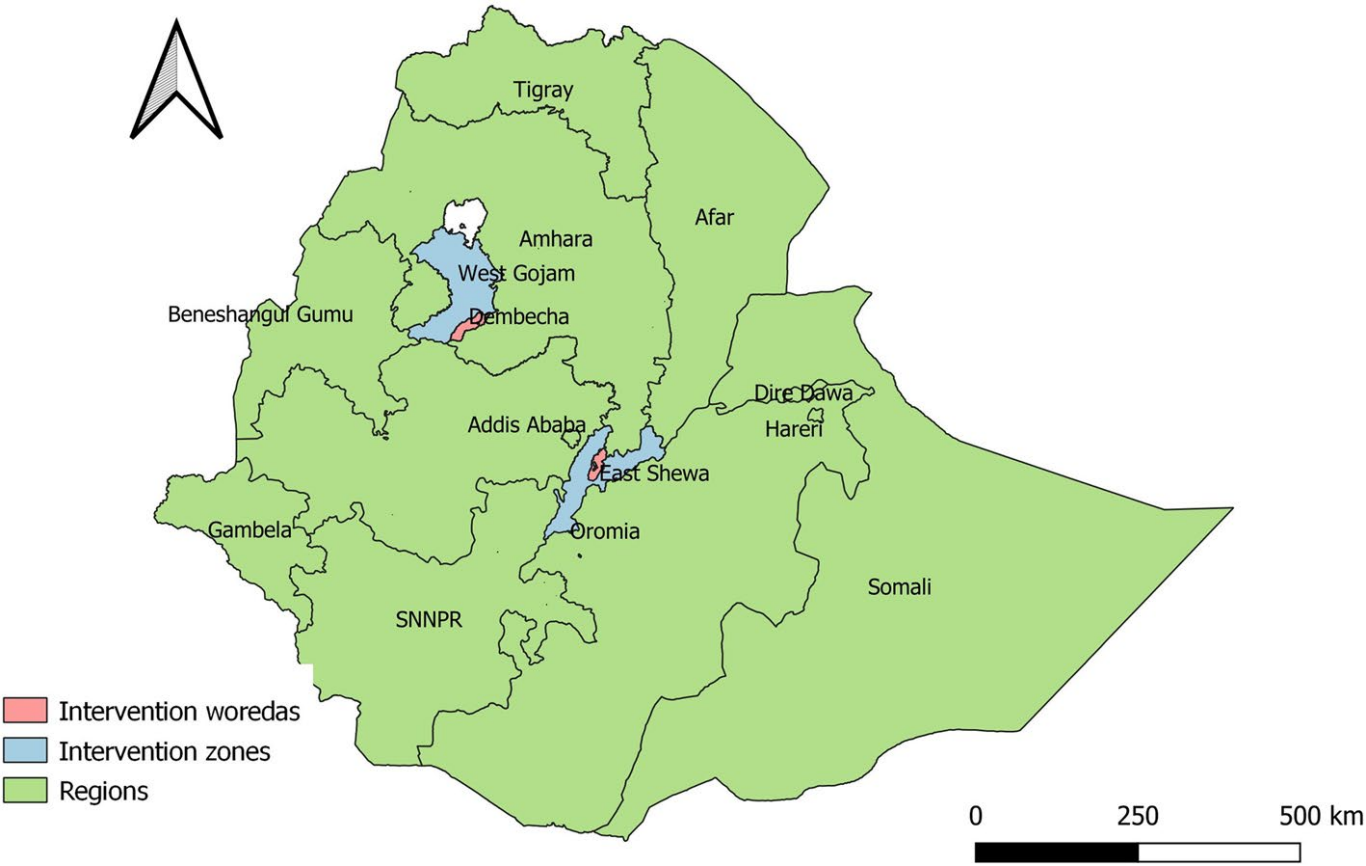
Map of community-based management of PSBI research implementation woredas during the COVID-19 pandemic in Ethiopia, 2022

This implementation research was conducted between November 2020-April 2022 in Dembecha and Lume woredas of Amhara and Oromia regions (Fig. 1) Dembecha is in the West Gojjam Zone of the Amhara region. The woreda town is 349 km north of Addis Ababa and 215 Kilometers South of Bahir Dar, the regional capital. It has 31 rural kebeles with poor electricity and road access. Based on the 2007 national census conducted by the Central Statistical Agency of Ethiopia [27], the woreda has a total population of 129,260, with 13.9% urban inhabitants. Thirty-one health posts (HPs) and six health centers provide primary health care services in Dembecha. Lume is located in East Shewa Zone of the Oromia region in the Great Rift Valley. The woreda capital, Modjo, is located 64 km east of Addis Ababa and 19 km east of Adama, the zonal capital. The 2007 national census [27] reported a total district population of 117,080, 33.06% of which are urban dwellers. The woreda has 35 rural kebeles with better access than Dembecha to health facilities, electricity, and roadways. 35 HPs and seven health centers provide primary health services.

## Methods

We employed narrative review, formative assessment, and facilitated stakeholder engagement to develop the management of PSBI IRLM. The work was designed to articulate determinants, choose implementation strategies, and link these through mechanisms to targeted implementation outcomes. In addition, during the implementation phase, we captured ongoing implementation challenges and adaptations of strategies using a tracker. We applied the Framework for Reporting Adaptations and Modifications to Evidence-based Implementation Strategies (FRAME-IS) [28]. We also identified metrics for implementation outcomes and process evaluation to measure the implementation of the strategies.

### Narrative review

**Fig. 2:**
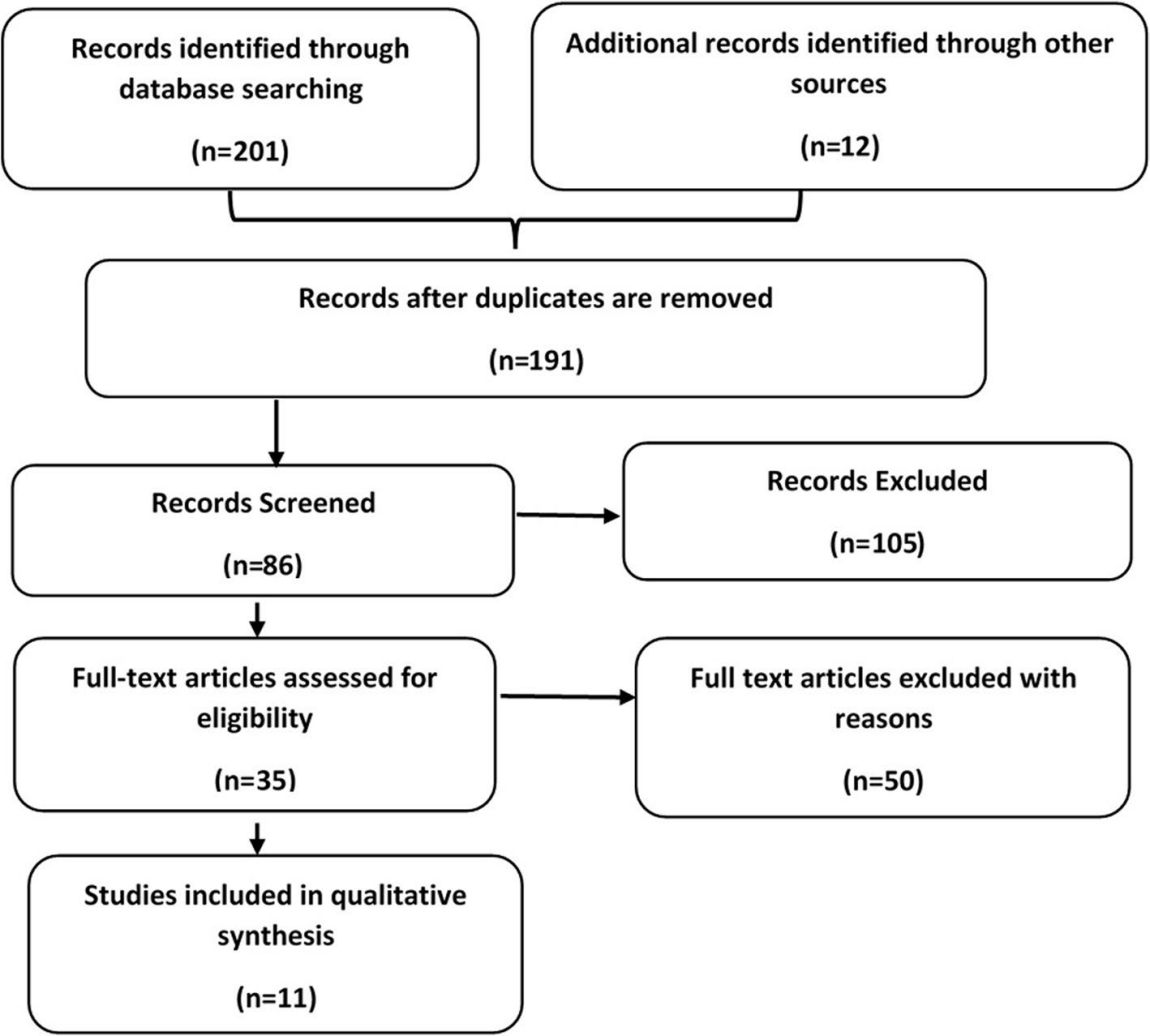
Review flow diagram

In April 2021, we conducted a narrative review of existing literature to quickly identify the overview of previously described barriers and facilitators of community-based management of PSBI implementation in Ethiopia. One author (GT) searched Google Scholar and PubMed for publications in English between 2012 to 2021, starting after the initiation of CBNC services in Ethiopia. Search terms used included: possible serious bacterial infection, community-based management of newborn illnesses, neonatal sepsis, uptake, utilization, barriers, facilitators, determinants, and Ethiopia. The title or abstract of the article was downloaded. The Preferred Reporting Items for Systematic Reviews and Meta-Analyses (PRISMA) diagram (Fig. 2) was used to select articles for this review. A narrative synthesis was used to extract themes of the barriers and facilitators to community-based management of PSBI implementation in Ethiopia. Eleven papers were included in the narrative analysis (five qualitative [19–23], four mixed [29–32], and two quantitative design [9, 33] studies) (see Additional file 1 for details of the characteristics of reviewed articles).

### Formative assessment

Guided by the RE-AIM (Reach, Effectiveness, Adoption, Implementation, and Maintenance) [34] framework and Consolidated Framework for Implementation Research (CFIR) [35], we conducted concurrent quantitative and explorative qualitative formative research in April–May 2021 in the two woredas. The goals were to understand the current implementation of community-based management of PSBI and implementation challenges to establish benchmarks, identify context-specific implementation gaps, and inform potential strategies to bridge these gaps.

We conducted a cross-sectional population-based household survey of 4,242 mothers who gave live birth 2–14 months before data collection and resided in the two woredas. Survey captured information including household and sociodemographic characteristics, experiences related to using maternal and newborn health services across the continuum of care, care-seeking for sick infants and children, and Knowledge and risk perception of COVID-19. A structured questionnaire translated into local languages (Amharic and Oromiffa) was used to capture the data using a web-based mHealth platform (SurveyCTO) using smartphones (Additional file 2). We also conducted surveys of 66 HPs and captured self-administered interviews with 79 HEWs available on the day of the visit. Surveys were based on an adapted tool (Additional file 3) from the WHO Service Availability and Readiness Assessment (SARA) [36]. They were designed to capture the availability of drugs, equipment, and knowledge and skills of HEWs.

We conducted 34 in-depth interviews of purposively selected respondents, including program managers (6), development partners (4), direct service providers (16), and community volunteers (8). The interview guide was designed using CFIR to understand what factors facilitate or hinder community-based management of PSBI implementation, including characteristics of the health system, intervention, individual, inner, and outer settings.

### Stakeholder engagement

We engaged stakeholders throughout the research process as a keystone of our implementation research in the selection, prioritization, and operationalization of tailored and appropriate implementation strategies to improve the adoption, implementation, and sustainment of community-based management of PSBI. For this purpose, we followed a rigorous and thoughtful development process to systematically plan for the implementation strategies by facilitating a national stakeholder consultation which expanded on adapted expert recommendations for implementing change (ERIC) protocol [37] employing a modified Delphi approach and woreda-level co-creation.

#### Consultations with stakeholders

Following the formative assessment and exploration of potential strategies from the literature, we developed an initial IRLM. Then, we conducted a national co-designing workshop on May 11–12, 2021. The workshop involved the Ministry of Health (MOH), National Child Health Technical Working Group, including program managers and development partners; it also included the National RMNCH-N Research Advisory Council Child Health and Immunization group, including their program managers, academics, researchers, and development partners. The workshop also included development partners active in neonatal care in Ethiopia. The workshop’s primary aim was to share the assessment findings and design tailored solutions to address implementation challenges.

On the first day, the research team presented an overview of implementation research and formative assessment findings, followed by a discussion on the barriers to community-based management of PSBI implementation. On the second day, the research team presented the draft IRLM, including the identified determinants and preliminary implementation strategies from the literature. Next, the team facilitated a group process of identification and prioritization of implementation strategies. The research team then populated the determinants and strategies of the IRLM raised through an iterative discussion with stakeholders and used the matrix to understand the conceptual links between determinants, implementation strategies, mechanisms, and outcomes.

#### Woreda-level contextualization and micro-planning

In the last week of May 2021 and the first week of June 2021, we conducted a woreda-level co-creation workshop with regional health bureaus, zonal health departments, woreda health offices, facility-level managers, service providers, kebele managers, and HEWs. Through the co-creation workshop, L10K and participants contextualized and validated the nationally co-designed implementation strategies, identified additional local contextual community-based management of PSBI implementation challenges that had not been captured during the national co-design phase, designed relevant strategies, and developed micro-plans to improve PSBI treatment implementation. All HPs and health centers in the two implementation woredas developed micro-plans. (See Additional file 4 for the detailed implementation strategies designed during the workshop).

### Iterative learning process and capturing ongoing challenges and adaptations of strategies

During program implementation, the project provided technical support to woredas to implement and monitor the adapted strategies. Throughout project implementation and process evaluation, we systematically captured ongoing implementation challenges and adaptation of the implementation strategies using the Framework for FRAME-IS [28]. We facilitated local communities of practice (CoP), learning visits, supportive supervision, and performance reviews to monitor the high fidelity of implementation strategies. In addition, we participated in monthly community-based management of PSBI CoP calls to share examples of work, including the IRLM, mapping strategies, standardized processes, approaches, methods, and metrics with the cross-country teams—including the teams in Kenya and India. The implementation strategies and IRLM were updated throughout the implementation process.

### Process evaluation

In October 2021, we conducted a process evaluation to measure the implementation fidelity of the adaptive implementation strategies to understand ‘what works and does not work’ by exploring and capturing ongoing implementation challenges and adaptations of strategies.

## Results

### Pathways of community-based management of PSBI adaptive implementation

Based on the literature findings, formative assessment, and stakeholder engagement, we developed the pre-implementation IRLM for two different contexts—Dembecha and Lume woredas (Fig. 2). We also recognized that while the evidence-based intervention (PSBI treatment when referral was not possible) was the same, contextual factors differed between the two areas. Therefore, the IRLM allowing for two different contexts was used.

### Determinants of Implementation

Multiple pre-existing challenges were identified (Table 1), with some differences and similarities across the two woredas.

**Table 1:** Barriers and facilitators of community-based management of PSBI when a referral is not possible during the COVID-19 pandemic

CFIR domains	Barriers and facilitators
Characteristics of the intervention	(-) Complexity of the intervention
	(-) Perceived scalability, feasibility for scale
Outer setting	(-) Mother’s fear of COVID-19 infection at health facilities
	✓ 58.9% of mothers in Lume and 25.2% of mothers in Dembecha reported fear of COVID-19 infection for themselves or their fetus during ANC, delivery, or seeking care for a sick child
	(-) COVID-19 response measures (restricted mass gatherings, limited mobility, and restricted public transportation)
	( ±) Geographic distance to higher-level facilities (-) Phase-out of partner support
	( +) NGO-delivered support
	(-) Community perceptions about newborn illnesses and sociocultural beliefs
	(-) Low functionality of WDAs
	✓ Lume: 65.5%; and Dembecha: 64.5% WDAs were active
	(-) Low community demand
	✓ Lume**:** 4.7% prevalence of sepsis; 11% prevalence of neonatal sepsis
	✓ 69.9% and 52.4% of mothers in Lume and Dembecha, respectively, received antibiotics for neonatal sepsis
	(-) State of Emergency and business shutdown in Amhara region (and shift of attention and resources)
Inner setting	(-) Supply and logistics management system
	✓ 68.6% and 51.6% of HPs stocked out for gentamicin 20 mg/ml in Lume and Dembecha, respectively
	(-) Strength of HEP
Individuals involved in the implementation	(-) Competence and confidence of HEWs
	✓ 40.5% and 33.3% of HEWs knew all signs of PSBI in Lume and Dembecha, respectively
	✓ There were 16 untrained HEWs on iCCM in Dembecha
	(-) Motivation of HEWs
	(-) Attendance/engagement of HEWs and PHCUs on PSBI treatment work
	(-) Competing priorities
	(-) Fear of COVID-19
	(-) HEWs workload and engagement in non-health activities
Process of implementation	(-) Integration of management of the PSBI program
	( +) Coordination, ownership, and stakeholder engagement

*ANC* Antenatal care, *iCCM* Integrated community-based management of common childhood illnesses, *HEW* Health Extension Worker, *HP* Health posts, *NGO* Non-governmental organization, *PHCU* Primary Health Unit, *PSBI* Possible serious bacterial infection, *WDA* Women Development Army

#### Intervention characteristics

From the narrative review, we identified the complexity of implementing the community-based management of PSBI intervention as a challenge. Our respondents also reported that the management of PSBI interventions was too complex for HEWs to perform, including identifying signs and symptoms of infection, assessing and classifying cases among newborns, and administering gentamicin injections. HEWs also perceived that the management of PSBI was too complex for them, given their current knowledge and skills. This barrier identified the need to address numerous factors along the care-seeking and caregiving continuum.

#### Inner setting

Both the formative assessment and narrative review identified the following as major barriers to the management of PSBI program in most HPs: inconsistent supply of essential drugs, poor logistics management systems, shortage of HEWs, the decline of the HEP performance in recent years, and a weak support system. We also identified differences in the magnitude of some factors, such as gentamicin stockout between Lume and Dembecha woredas— 69% of HPs in Lume and 52% of HPs in Dembecha for gentamicin 20 mg/ml on the day of data collection.

#### Outer settings

COVID-19 pandemic posed an unprecedented challenge to delivering PSBI treatment in the first 2–3 months since the first COVID-19 case in Ethiopia in March 2020. The management of the PSBI program was interrupted due to staff panic and the closure of facilities. More women in Lume compared to Dembecha reported COVID-19 as a barrier to seeking care for their sick children. Maternal fear of contracting COVID-19, infecting their fetus during ANC or their child during either delivery or while seeking postnatal care, was higher in Lume than Dembecha.

The formative assessment identified a high prevalence of newborn illness in rural communities and low uptake of integrated community case management (iCCM) of childhood illnesses and newborn care, particularly for sick young infants (SYI). A higher proportion of mothers who resided in Dembecha (11%) compared to Lume woredas (5%) reported symptoms of severe neonatal infection in their infants. Communities in Dembecha demonstrated lower care-seeking behavior compared to Lume woreda, with only 52% of mothers receiving antibiotics for neonatal sepsis there compared to 70% of mothers in Lume. Most women sought care for their SYI at health centers and bypassed the HPs. Additionally, the following pre-existing factors were identified as external barriers to the delivery of PSBI treatment: phase-out of partner support, low community care-seeking behavior, negative community attitudes toward services for neonatal illness and PSBI, lack of community trust in HEWs’ capacity to manage PSBI, and low functionality of organized female community volunteers called Women Development Armies (WDAs).

#### Characteristics of individuals

The following characteristics were identified as barriers to HEW management of PSBI implementation: lack of competency and confidence in their PSBI knowledge and skills; lack of training, mentoring, and coaching; low motivation, attendance, and engagement regarding PSBI care implementation; competing workload and engagement in non-health activities; and fear of contracting COVID-19. In Dembecha, HEW knowledge of all PSBI signs was lower than in Lume, with 33% and 41%, respectively. Likewise, a higher number of HEWs untrained in the basics of PSBI were identified in Dembecha compared to Lume.

*Process*: The formative assessment, as well as the stakeholder consultation, identified the following implementation process barriers to PSBI care: poor integration of management of PSBI services in the woreda health system, weak coordination, ownership, and stakeholder engagement, and a weak culture of learning.

### Implementation strategies

Previous implementation research on the management of PSBI when referral was not feasible conducted in Ethiopia, Malawi, Nigeria, Bangladesh, Pakistan, and India identified several strategies critical to supporting effective implementation at the individual, community, facility, and systems levels. At the individual and community level, these included: training health care workers, in-field technical support, performance reviews [8], and community engagement [8–16, 38]. At the facility and systems levels, strategies included: strengthening the identification and assessment of young infants for illness, improving supply provision, strengthening supply chain management [8, 38], standardization of treatment protocols at the health center and HP levels [8], policy dialogue, presence of state-level sensitization workshop, and establishment of a technical support unit.

During the stakeholder engagement, based on the formative assessment and narrative review findings, we grouped major management of PSBI implementation challenges that existed before COVID-19 and were exacerbated by the pandemic into the following categories: 1) low competency, confidence, and motivation of HEWs; 2) weak primary health care system; 3) suboptimal community engagement; 4) other systemic factors (i.e., HEW workload and engagement in non-health activities); and 5) weak coordination and integration. We also presented results from the literature review and formative assessment. In consultation with stakeholders, we adopted and adapted a number of the strategies from the literature and developed new ones, including reflecting on COVID-19 -related challenges to enhance the adoption, acceptability, and high-fidelity implementation of community-based management of PSBI interventions, with the aim of further integration and scale up.

As presented in Table 2 below, we identified implementation strategies at various levels. At the individual level, we identified on-site coaching and training of HEWs by PSBI/iCCM-trained focal mentors from health centers as strategies. At the facility- and systems level, we identified the following strategies: coordinated referrals between HPs and health centers; the establishment of a technical support unit to facilitate implementation across the area; strengthening of the supply chain, support system, and links; integration of management of PSBI implementation in the district health system work stream: and integration of COVID-19 and routine services.

**Table 2:** Community-based management of PSBI implementation strategies during the COVID-19 pandemic in Ethiopia, 2021

Determinants	Strategies from literature	Management of PSBI implementation strategies
**Low competency and confidence of HEWs (inner)** • Lack of training and mentoring • Low competency and self-efficacy of HEWs	• Training of health care providers • Standardization of treatment protocols at the health center and HP levels • Active case finding • Integration of COVID-19 guidelines and materials into the IMNCI/PSBI training curriculum • Recruitment of community health volunteers (CHVs) • HEWs incentives	• Coach and train HEWs on-site (case demonstrations and scenarios-based coaching) • Train HEWs and their supervisors on PSBI treatment • Strengthen the implementation of home visits by WDA leaders and HEWs • Leverage the existing HEW workforce and activate engagement of HEWs
**Weak primary health care system (inner)** • Weak support system and low engagement of the PHCU • Inefficient service and health system linkages • Service availability and access (HP closure and declining HEP) • Weak M&E and learning systems • Erratic supply (and poor forecast and requisition) • Non-resilient PHC and negative impact of COVID-19 • No/weak motivation mechanisms	• Supportive supervision • Community-facility referral link • Quarterly review meetings with the responsible stakeholders • Data audit and feedback • National protocol and guideline development • Community-based surveillance of pregnant women and birth; home-based postpartum visits and identification of SYIs and treatment or referral • Strengthen implementation of home visits by WDA leaders and HEWs • Referral system strengthening (communication, referrals) • Strengthening data capture • Review and adaptation of digital platforms to capture data on the management of PSBI activities • Health system strengthening (supply chain management strengthening) • Supply provision • Advocacy and training for supply chain strengthening and management	• Activate engagement of PHCUs, HEWs, and WDAs • Strengthen regular PHCU-level integrated PRCMM and support (focused on PSBI intervention implementation challenges and skill gaps) • Strengthen health center and HP links (e.g., assign a focal person for PSBI treatment) • Introduction of management of PSBI module into eCHIS (clinical decision support tool) • Strengthen implementation of home visits by WDA leaders • Integrate COVID-19 and routine services • Strengthen facility-level IPC practices • Monitor the regional COVID-19 situation • Strengthen supply chain and logistics management system (e.g., strengthen resource quantification and forecasting both at HP and facility level; follow the use of bin cards) • Advocacy for procurement of gentamicin • Redistribution of gentamicin 20 mg/ml
**Suboptimal community engagement and outreach (outer)** • Low functionality of WDAs • Low trust in HEWs’ capacity to manage PSBI Community perceptions about newborn illnesses	• Community engagement • Community mobilization and linkages • Consultative meetings with stakeholders • Working with Women Development Army • Community education • Community sensitization and awareness campaigns	• Community mobilization and links • Community sensitization and SBCC activities • Work with kebele managers to make WDA structure functional
**Other systemic factors (inner)** • HEW workload and engagement in non-health activities are too high		• Introduce regular kebele-level multi-sectoral meetings
**Weak coordination and integration (outer)** • Suboptimal ownership • Poor integration of management of PSBI service delivery and support	• Policy dialogue and consultative process • Technical support units for technical back-up • Memorandum of understanding between technical support unit and stakeholders that defines roles and responsibilities • Alignment with national protocols • County-level sensitization, buy-in, and support • Engagement of Ministry of Health (MOH) leadership • Non-government organization-delivered support	• Participatory design and implementation with national and district-level stakeholders • Strengthen woreda-level integrated performance reviews • Monitor visits from the woreda health office and the IR Project • Integrate PSBI treatment into the pre-existing systems for review and accountability at each health system level • Organize technical support units • Integration of COVID-19 and other services • Conduct advocacy meetings (with woreda administration for free ambulance service for SYI emergencies) • Provide on-site orientation for kebele managers to prioritize CBNC activities

CBNC Community-based newborn care, *CHV* Community health volunteer, eCHIS electronic community health information system, *IMNCI* Integrated management of newborn and childhood illnesses, *HEW* Health Extension Worker, *HEP* Health Extension Program, *PHCU* Primary Health Care Unit, *PRCMM* Performance review and clinical mentoring meeting, *PSBI* Possible serious bacterial infection, *SYI* Sick young infant, *WDA* Women Development Army

### Implementation mechanisms

**Fig. 3:**
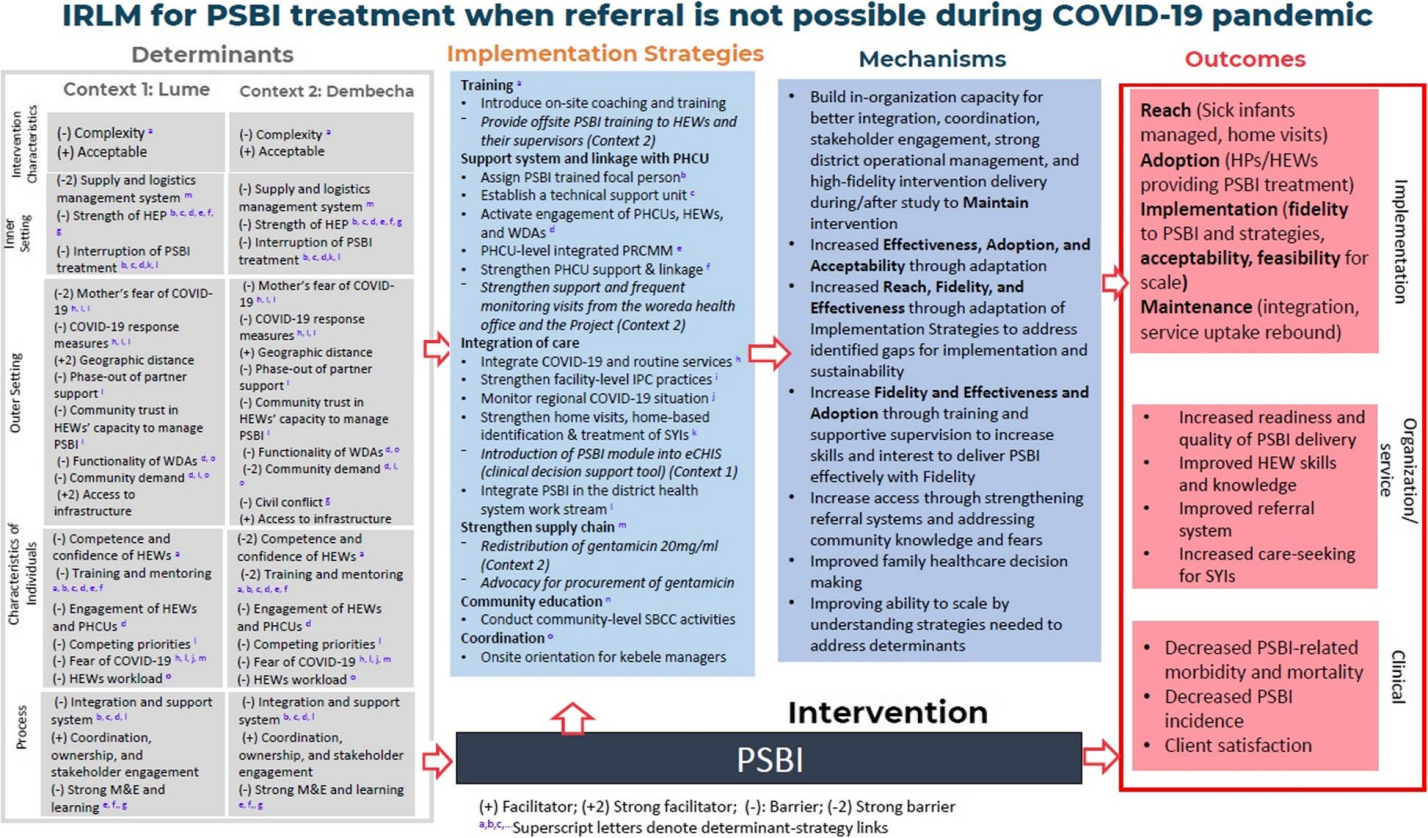
The IRLM for community-based management of PSBI when a referral is not possible during the COVID-19 pandemic in Ethiopia, 2021

Using the IRLM, we described the mechanisms of action that the proposed strategies would operate to influence the desired outcomes. For example, building the organization's capacity for better integration, coordination, and stakeholder engagement would lead to strong district-level operational management and high-fidelity intervention delivery during/after study to maintain strategies. Adoption of the strategies, training and supportive supervision increased skills and interest in delivering PSBI treatment effectively and with higher fidelity. It also strengthened referral systems, addressed community knowledge and fears while increasing fidelity, effectiveness, and adoption (Fig. 3).

### Implementation outcomes

At the start of the project, we identified implementation outcomes, proximal impacts of the strategies, and their mechanisms using RE-AIM [34] and Proctor et al.’s taxonomy of implementation outcomes [39]. Previous studies [10, 14] defined metrics as *Reach:* number of newborns identified and visited by community health workers, percentage of treatment coverage, and percentage of referral refusal; *Effectiveness:* recovery, and case fatality rate; *Fidelity:* percentage of patient adherence to antibiotic treatment, percentage of patients who received follow-up care, percentage of classification errors, and percentage of patients who received correct antibiotic; *Acceptability:* number of parents/family who refused referral but accepted community-based PSBI treatment, barriers and facilitators of acceptability and care-seeking, percentage of PSBI cases who accepted the referral, and rate of adherence to outpatient treatment. We developed a matrix of indicators and data sources for adaptive implementation of PSBI treatment during COVID-19 in Ethiopia (Table 3). This table includes our metrics: reach, effectiveness, adoption, feasibility, fidelity, acceptability, and maintenance.

**Table 3:** Matrix of indicators and data sources for adaptive implementation of community-based management of PSBI program during the COVID-19 pandemic in Ethiopia, 2021

RE-AIM framework	Metrics	Data sources
**Reach:** *The degree to which an intervention-eligible population receives it (Coverage)*	• # of sick young infants managed for PSBI (% infants eligible who receive PSBI care) • # of sick newborns and young infants managed • Proportion of SYI referred	Facility iCCM/PSBI register review
**Effectiveness:** *The impact of an intervention on targeted outcomes*	• Improvement in % of mothers/caretakers of SYIs who seek care from an appropriate provider • % of mothers’/caretakers’ adherence to treatment advice (caretaker level) • Care provider adherence to PSBI management	Before-after household surveys Facility iCCM/PSBI register review
**Adoption:** *Intention, initial decision,* or action to employ new intervention (uptake)	• # of facilities providing PSBI treatment services when a referral is not possible • # of facilities and community-based providers trained in PSBI treatment	Program monitoring data
***Feasibility:**** the extent to which a new strategy can be successfully used or carried out within a given setting*	• Percent of supplies in stock and percent of re-supply	• Process evaluation (facility assessment)
**Fidelity:** *Degree to which an intervention or strategy was implemented as it was designed in an original protocol, plan, or policy (or as adapted)*	• Ongoing implementation challenges • Providers' adherence to PSBI case management protocol • Implementation strength score ○ *% of HEWs on average trained/mentored on PSBI treatment* ○ *Mean percentage of materials/ equipment available* ○ *Mean percentage of supplies available* ○ *% of HEWs supervised on PSBI treatment* ○ *% of HPs participated in the PHCU level PRCCM meetings in the last six months* ○ *% of HPs facilitated kebele-level multi-sectoral meetings at least once in the last six months* ○ *% of HPs facilitated awareness creation meetings at the community level* • % of mothers’/caretakers’ adherence to treatment advice	Process evaluation (facility assessment, interview with service providers and program managers, facility iCCM/PSBI register review) Program monitoring data Before-after household surveys
**Acceptability**: *Perception among stakeholders that intervention is agreeable*	• Barriers and facilitators to uptake of PSBI treatment services • % of mothers/caretakers of SYI who seek care from an appropriate provider • Parent/caregiver acceptance of PSBI treatment	Process evaluation (interview with service providers) Before-after household surveys
**Maintenance:** *Extent to which an intervention is maintained or institutionalized in a setting (Sustainability)*	• Implementation strategies incorporated with the woreda work stream and integration • Trends in service uptake and rebound • Feasibility of the approach for national scale-up	Process evaluation (facility assessment, interview with service providers and program managers, facility iCCM/PSBI register review)

*iCCM* integrated management of common childhood illnesses, *SYI* Sick young infant, *PHCU* Primary Health Care Unit, *PRCCM* Performance review and clinical mentoring meeting, *PSBI* Possible serious bacterial infection

The implementation outcomes from the RE-AIM process evaluation are presented below. Most HPs conducted different community dialogues with kebele leaders, WDAs, and kebele-level multi-sectoral meetings to create demand for PSBI treatment. The iCCM trained personnel from the under-five clinic of health centers, health center heads, and woreda child health officers were assigned to serve as a technical support unit and provided on-site training and coaching to HEWs through supportive supervision; facilitated performance reviews; coordinated referrals and provided feedback to HEWs; identified implementation challenges; and supported community mobilization. The program monitoring data showed that almost all HPs received support from their catchment PHCUs at least once from April to September 2021. Out of 107 HEWs available, 87 were trained or mentored on-site on iCCM in the last six months. The data from the HP assessment show that 77 HEWs (72%) from 42 HPs reported participation in at least one PHCU level PRCMM in the last six months (Table 4).

**Table 4:** iCCM program implementation intensity during baseline, mid-term, and end-line survey periods, October 2021

Measures	April 2021	October 2021
% of HEWs trained/mentored on iCCM/PSBI	-	82.6
% of HEWs supervised on iCCM/PSBI	-	71.8
% of HPs participated in the PHCU level PRCCM meetings in the last six months	-	66.2
% of HPs facilitated awareness creation meetings at the community level	-	96.9
% of HPs facilitated kebele-level multi-sectoral meetings at least once in the last six months	-	46.1
% of WDAs that are functional (i.e., meet with HEWs, report activities to HEWs, do home visits, and identify sick newborns) in the last three months	65.1	60.9
% of HPs treated sick young infants in the previous six months	35.9	80.0
Total SYI cases seen (September 2020-March 2021 vs. April 2021-September 2021)	149	292

*iCCM* integrated community-based management of common childhood illnesses and newborn care, *HEW* Health Extension Worker, *HP* Health post, *PHCU* Primary Health Care Unit, *PRCMM* Performance review and clinical mentoring meeting, *PSBI* possible serious bacterial infection

From April-September 2021, HEWs made 5,465 home visits, identified 645 newborn infants, and treated 292 sick young infants. This was an increase of 49% from 149 cases during September 2020-March 2021. Accordingly, fifty-one (80%) HPs treated at least one sick young infant in the previous six months, significantly improving from 36% in the last six months.

### Adaptations and modifications of strategies

During implementation, we tracked ongoing implementation challenges and adaptive strategies. We used the IRLM framework to capture these emerging or persistent challenges and adapted strategies to articulate the mechanisms of action and link them to outcomes (Fig. 3).

During the implementation, adaptive implementation strategies were also incorporated, including facilitating the redistribution of gentamicin 20 mg/ml, intensifying the support and monitoring visits, and increasing offsite PSBI treatment training of HEWs and their supervisors at HPs in Dembecha. Due to the civil war in the northern part of the country, a regional emergency decree was issued on October 31, 2021, halting the regular services of public institutions. This subsequent shift in attention and resources away from the management of the PSBI program emerged as a challenge in Dembecha. We provided one round of supervision when the national and regional situations improved. The district health office conducted monitoring visits, performance reviews, and corrective measures to reestablish prior support for community-based management of PSBI services and strengthen their implementation.

In the Lume district, NGO support for maternal and child health was identified as a facilitator for the management of PSBI service during implementation. In addition, the MOH introduced a digital version of the PSBI/iCCM module into the electronic Community Health Information System (eCHIS) in Lume in August 2021. The eCHIS was developed to replace the paper-based information system, a family folder containing household information, registration for incoming and leaving household members (member registration), data regarding service provision, and referral services rendered. The eCHIS incorporated job aids to support service provision, improve quality of care, and improve data use for decision-making. As such, digitization of the PSBI/iCCM treatment module aided HEWs as a decision support tool. It also facilitated digital referral, identification, and registration of newborns and SYI in the app during household visits, leading to improved delivery of community-based management of PSBI services (Table 5).

**Table 5:** Adaptations and/or modifications of community-based management of PSBI implementation strategies during the COVID-19 pandemic in Ethiopia, 2021

Strategy	Adaption/ modification	Timing of modification (pre, during, end)	Planned/ ad hoc	Who decided/ participated	Reason for modification	Modifications	Evidence of effect
Community-based management of PSBI training (Dembecha)	Organized offsite training for HEWs and their supervisors	During implementation (December 2021)	Planned	Implementation team	To improve appropriateness of the strategy (greater # of HEWs not trained on basic PSBI treatment and restrictions on gatherings due to COVID-19 lifted)	Form (mode of delivery) and content	Improved competency in pre-post training evaluation score
Supportive supervision (Dembecha)	Intensified support system	During implementation (November–December 2021)	Planned	Implementation team	To address the contextual challenge of a shift in resources and attention following the SOE and business shutdown	Change in the form (intensified the support system)	More frequent support improved the performance of HEWs [40]
Supplies (Dembecha)	Redistributed gentamicin 20 mg/ml	During implementation (July 2021)	Planned	Implementation team	To address a shortage of supplies	New strategy	The supply of drugs was a critical component of PSBI treatment implementation
iCCM module incorporated into eCHIS (Lume)	Digitized PSBI treatment protocol	During implementation (August 2021)	Ad hoc	MOH	To test the digital PSBI treatment protocol	New strategy	Electronic clinical decision algorithms improved the management of sick children in primary care [41] The eCHIS app potentially facilitated the identification and registration of newborns and SYIs, referral, and clinical decision

*eCHIS-electronic* Community Health Information System, *iCCM* Integrated community-based management of common childhood illnesses, *HEW* Health Extension Worker, *MOH* Ministry of Health, *PSBI* Possible serious bacterial infection, *SOE* State of Emergency, *SYI* Sick young infant

## Discussion

We successfully employed implementation research frameworks in designing, implementing, and evaluating the adaptive implementation of PSBI treatment during COVID-19 in Ethiopia. The RE-AIM and CFIR frameworks were used to design the COVID-19 pandemic that needed to be addressed to improve the reach, adoption, fidelity, and uptake of community-based management of PSBI services. In addition, we populated an initial IRLM. We used the framework to meaningfully engage stakeholders to systematically develop, identify, categorize, and prioritize implementation strategies to address identified and newly emerged barriers. We found that using an increasingly used framework, which combines CFIR and RE-AIM using concept mapping, effectively identifies strategies by integrating existing evidence and stakeholder engagement and expertise.

The learnings demonstrated the unprecedented challenges of the COVID-19 pandemic, and the pre-existing health system-level barriers exacerbated the fragile community-based management of PSBI in Ethiopia. Previous studies revealed significant service disruptions in outpatient care and child vaccinations [42]; a decline in clinic attendance and hospital admissions of children [43, 44]; and delayed health-seeking for child health services [45, 46] during the pandemic indicating fear of COVID-19 might intensify delays and reduce access to newborn care. In addition to COVID-19, the following pre-existing factors proved to be the most formidable challenges in the management of PSBI implementation. To address these challenges, we identified the following as key mitigating implementation strategies: the integration of PSBI treatment in the district health system work stream, the integration of COVID-19 services with primary health care services, continuous on-site coaching and training of HEWs, the creation of a robust support system from health centers and woreda health offices, the use of participatory design and implementation methods, the strengthening of the supply chain system, and the presence of community-level SBCC activities. These approaches 1) effectively engaged community volunteers, HEWs, and the primary health care system; 2) recovered services interrupted due to COVID-19; 3) improved community awareness about the iCCM/PSBI treatment availability; and 4) integrated PSBI treatment with the PHCU and woreda workstream for sustained implementation and scaled up.

Using implementation research methods to inform the evidence synthesis using narrative review and formative assessment helped us better identify the challenges and provide an initial understanding of the conceptual links between determinants, implementation strategies, mechanisms, and outcomes. The use of concept mapping is essential in informing the choice of implementation strategies and is associated with more effective implementation [26, 37]. Stakeholder engagement and co-creation have been identified as key steps in designing and implementing complex interventions such as treatment for PSBI [26, 47]. We also found that these strategies helped us gain insights and clarity to develop the PSBI treatment IRLM. Furthermore, our iterative learning and deliberate capturing of ongoing challenges and strategies modifications were critical to understanding and conceptualizing the links between determinants, implementation strategies, mechanisms, and outcomes.

Throughout our implementation research, we employed IRLM and expanded the adapted ERIC protocol and concept mapping technique to engage stakeholders to gain insights and clarity for developing the PSBI IRLM. This allowed for the systematic planning and application of the community-based management of PSBI implementation strategies that consider determinants, mechanisms, and outcomes. Consequently, we bridged implementation gaps by selecting strategies that improved the implementation process, increased adoption, and sustained effective intervention uptake [26]. Current IR theories and prior research lacked consideration of ground-level stakeholder knowledge and preferences. As a result, implementation strategy development and selection challenges surface, which affect the adoption and application of evidence-based interventions [26, 37].

The IRLM enabled the development of adaptive implementation strategies based on an in-depth understanding of the barriers and facilitators. The application of the framework also allowed us to identify implementation challenges, strategies, and mechanisms of action for adaptive implementation of PSBI treatment to increase implementation fidelity, reach, and adoption. Furthermore, these adaptive implementation strategies elucidated changes in the care-seeking behavior of mothers for their sick young infants. IRLM helped guide the measurement of the activities, intermediate outputs, and outcomes and synthesizing lessons from implementation.

During implementation, we tracked ongoing implementation challenges and adaptive strategies, using the IRLM framework to conceptualize the mechanisms of action and outcomes. We also employed this framework to document the implementation learnings and share them with the broader community. This allowed us to examine what happened during the implementation and accurately report the hypothesized relationships we observed. For instance, the digital version of the iCCM module, which is a mobile health (mHealth) solution for HEWs that incorporates job aids— to counsel mothers on essential newborn care and educate them on danger signs of PSBI, assess newborns for possible signs of infection, diagnosis, treat and counsel mothers to adhere to treatment protocols—improved the delivery of PSBI treatment. Specifically, it facilitated identifying and registering newborns and sick young infants and aided clinical and referral decisions and adherence to PSBI treatment protocols. This is in contrast to the responses from the data in Dembecha, where the iCCM module was not incorporated into eCHIS and where few or none of the above advantages from eCHIS were reported. At the same time, close monitoring and timely corrective measures to address emerging contexts like the effect of civil war are critical.

We continued using this framework to share examples of our work in the cross-country community of practice with the community-based management of PSBI Kenya and India teams to cross-map implementation strategies, adaptations, mechanisms of action, and implementation outcomes. We also explored efficient ways to capture contextual factors to build on our IRLM to inform the management of PSBI work at scale. This has informed our continued work across country projects to identify areas that could benefit from standardized processes, approaches, methods, and metrics.

Despite our efforts of exploring all possible challenges, co-designing and implementing strategies with active engagement of stakeholders, capturing ongoing challenges, and adapting strategies throughout the implementation period, we might not include all possible barriers affecting the community-based management of the PSBI program in the model. In addition, some of the determinants of this study may have been affected by social desirability and recall biases which would, in turn, affect the logical links between each component of the IRLM. Though the IRLM provides formats for a standard IR study that include multiple contexts and comparative implementation, it doesn’t explicitly show how to synthesize and report implementation outcomes across various contexts to compare variations in implementation processes and influences across contexts for understanding what works for whom, and how [48].

## Conclusions

In conclusion, we found that the IRLM used in this research helped us organize the formative work and facilitate stakeholder engagement to specify the conceptual links between the implementation challenges, strategies, mechanisms of action, and outcomes to design and strengthen the management of the PSBI program under complex contextual factors, including COVID-19, as well as an existing health system and community barriers. The capturing of ongoing implementation challenges and document modifications of strategies during implementation allowed us to update the IRLM to provide better generalizable knowledge on adapting and implementing management of PSBI in similar and different settings.

The participatory design and implementation of adaptive COVID-19 strategies effectively maintained the quality delivery of PSBI treatment during the pandemic. These approaches effectively engage community volunteers, HEWs, and the primary health care system and better integrate PSBI treatment for sustained implementation and scale up. Continuous support and feedback systems, continuous supply of essential drugs, and strategies to alleviate HEWs’ workload to deliver quality services should be prioritized.

## Supplementary Information

**Additional file 1.**

**Additional file 2.**

**Additional file 3.**

**Additional file 4.**

**Additional file 5.**

**Additional file 6.**

## Data Availability

The dataset used and analyzed during this study is included as supplementary information to this article (Additional files 5 and 6).

## Abbreviations

CBNC: Community-based newborn care
CFIR: Consolidated Framework for Implementation Research
COVID-19: Coronavirus
eCHIS: Electronic Community Health Information System
FRAME-IS: Framework for Reporting Adaptations and Modifications to Evidence-based Implementation Strategies
ERIC: Expert Recommendations for Implementation Change
HEP: Health Extension Program
HEW: Health Extension Worker
HP: Health post
iCCM: Integrated community case management of childhood illnesses and newborn care
IRLM: Implementation Research Logic Model
KOICA: Korea International Cooperation Agency
L10K: Last Ten Kilometers Project
MOH: Ministry of Health
PHCU: Primary Health care Unit
PRCMM: Performance review and clinical mentoring meeting
PSBI: Possible serious bacterial infection
RE-AIM: Reach, Effectiveness, Adoption, Implementation, and Maintenance
SOE: State of Emergency
SYI: Sick young infant
UNICEF: United Nations Children's Fund
USAID: United States Agency for International Development
WDA: Women Development Armies
