# Supplementary material for: Using the Implementation Research Logic Model to design and implement community-based management of possible serious bacterial infection during COVID-19 pandemic in Ethiopia
Source: BMC Health Serv Res. 2022 Dec 13;22:1515. doi: 10.1186/s12913-022-08945-9 (PMC9745284; doi:10.1186/s12913-022-08945-9)
Supplement: Supplementary file 1 — Additional file 1. [file 12913_2022_8945_MOESM1_ESM.docx]

Additional file 1: Study characteristics included in the narrative review and barriers and facilitators for uptake of PSBI treatment in Ethiopia, 2021

| **Study ID** | **Design** | **Objective** | **Settings and population** | **Main findings** | **Sample size** |
| --- | --- | --- | --- | --- | --- |
| Tareke 2020 [16] | Qualitative study | Exploration of facilitators of and barriers to the uptake of PSBI | Debre Libanos woreda, Oromia  Women who gave birth within 2 months, HEW, health workers, religious leader, kebele chairman, and other community members | **Barriers**   - Perception of no treatment; perception of non-severity and self-resolution; belief in the healing power of traditional medicines - Awareness about the availability of the service at the HP and the unavailability of HEWs at HPs during working hours - Socio-cultural and religious beliefs - The functionality of WDAs - Shortage of HEWs; residence of health extension workers; health workers’ commitment - Budget constraint   **Facilitators**   - Availability of trained human; resources equipped human resource - Availability of monthly PRM, supervision, monitoring, and evaluation of activities; availability of logistics [medical supplies and job aids] | 5 in-depth interviews (IDIs), 7 key informant interviews (KII), and 4 focus group discussions (FGDs) |
| Gebremedhin 2020 [32] | Cross-sectional household survey | Utilization of CBNC and associated factors of sick young infants | Geze Gofa woreda, SNNP  Women who had their newborns recently | Overall utilization of CBNC was 37.5%;  **Associated factors**: elementary school, college and above, farmer women, women in the lowest and middle quantile of wealth status, and those whose preference was visiting hospital only when they faced any signs of danger | 371 |
| Gebretsadik 2020 [17] | Qualitative study | Community acceptance and utilization of the CBNC services and enablers and barriers to HEWs’ services provision | Hawassa University HDSS, 4 districts, Sidama Zone  mothers, HEWs, their supervisors, and coordinators | **Acceptance**: services provided by HEWs at the community level for mothers and their children are highly appreciated and recognized.  **Performance of HEWs**: better in ANC, but PNC and community-based neonatal care were reported to be insignificant.  **Enablers:** positive attitude and smooth relationship with the community, kebeles leaders, WDA, and mothers  **Challenges and opportunities:** workload, road inaccessibility, poor supervision, inadequate drugs and equipment supply, shortage of manpower and budget at HPs, distance and topography factors of homes from HPs making the visits more difficult, negligence by HEWs due to long time services without promotion and benefits, etc. | **IDIs**: 8 HEWs, 8 program coordinators and supervisors,  4 **FGDS** with mothers with <12 months children (n=33) |
| Asfaha 2020 [19] | Qualitative study | Understand household care-seeking and decision-making strategies for PSBI symptoms | 2 woredas in Amhara region | key themes: maternal responsibility of the newborn; maternal decision-making; environmental, hygiene, and nutrition practices as drivers of illness; illness conceptualization based on complexity; and care-seeking a trajectory | 51 (mother, father, and household members); 14 community groups; 17 symptomatic groups |
| Bayray 2019 [28] | IR (mixed research) | Acceptance, practices, challenges, and opportunities of the SYI referral (caregivers’ perspective) | Raya Alamata and Raya Azebo woredas  Young infants aged 0-59 days with PSBI | 144 (69.2%) of those cases, main reason to decline the referral for 35 (54.7%) of the families was that “no one was to accompany the infant to the next level of care”. Distance from the referral hospital, lack of companion, and lack of transport as one factor that prevented them not to accepting the referral recommendation | 854 PSBI cases |
| Abdella 2019 [29] | Mixed methods (record review & IDIs with service providers) | Assess the referral adherence rate of caretakers of sick young infants and the barriers and facilitators to adherence | 5 zones of Ethiopia | Of the 145 caretakers who accepted the referral, 88% adhered to the referral  **Factors for referral adherence**: providing information on the severity of illness; spouse’s occupation; perceived severity of illness of infants, age of the sick infant, education level of the caretaker, support from caretaker’s spouse, prior experience of referral, and access to referral facilities.  **Healthcare providers’ side facilitators**: provision of information about the seriousness of the illness and the available quality of care at referral facilities to caretakers, psychosocial and physical support for mothers, and provision of a referral slip; communication between HPs and health centers was perceived as poor, despite formal meetings. | 145 PSBI referral cases  HEWs and health center staff |
| Gebremedin 2019 [30] | A case study evaluation design (mixed methods) | Evaluate the process of the CBNC program implementation | Geze Gofa district, SNNP | The overall level of the implementation process of the CBNC program was 72.7%, to which maternal satisfaction, availability of resources, and healthcare provider's compliance with the national guideline contributed 75.0, 81.0, and 68.0%, respectively.  Stock out of essential drugs and medical equipment; PSBI not treated according to the national guidelines, and poor identification of neonatal sepsis cases  **Factors associated with maternal satisfaction**: trading occupation and low wealth status | 321 mothers; 27 direct case observations, six-month document reviews, and 14 KIIs |
| Tareke 2020 [18] | Qualitative study | Explore community member’s perceptions, experiences, and health-seeking behavior toward newborn illnesses | Debre Libanos woreda. Oromia  Women who gave birth within 2 months, HEW, health workers, religious leader, kebele chairman, and other community members | Community members locally diagnose such as sunburn, evil eye, kichitat, megagna, berd, enlarged/dropping of uvula, and common cold  Primarily prefer traditional medications to manage the illnesses; clients seek health care for these newborn illnesses late. | 5 IDI, 7 KII, 4 FGDs |
| Onarheim 2020 [20] | Qualitative study | Explore mothers’ roles in decision making and strategies in care-seeking for sick newborns | Butajira HDSS, SNNP  IDIs and FGDs with primary care-takers who had experienced recent newborn illness or death, health care workers, and community members | Choices about whether, where, and how to seek care for ill newborns were made through cooperation and negotiation among household members; mothers considered the ones that initially identified or recognized illness, but their actual opportunities to seek care were bounded by structural and cultural constraints; mothers limited bargaining power, contained by financial resources and gendered decision-making, shaped their roles in care-seeking;  Three strategies identified that mothers took on in decision making for newborn illness: (a) acceptance and adaptation (to the lack of options), (b) negotiation and avoidance of advice from others, and (c) active care-seeking and opposition against the husband’s or community’s advice. | 14 IDIs & 7 FGDs |
| Leul 2021 [6] | Implementation research (longitudinal) | Assess the feasibility of the simplified regimen | Raya Alamata and Raya Azebo Woredas | Challenges encountered: poor pregnancy surveillance, PNC home visits; poor facility linkages; poor supervision; suboptimal quality of HEWs assessment; HEWs workload; poor healthcare-seeking behavior of mothers; closure of HPs during working hours | 854 SYIs |
| Mathewos 2019 [31] | Mixed methods (routine program databases and desk review and after-action reviews with stakeholders) | Measure the strength of the CBNC program implementation | CBNC intervention zones | Trained service providers were available in all HPs and 91% (95 CI: 90-92) of HPs had the essential drugs, amoxicillin, and gentamycin for community case management of sick newborns on the day of the visit; 32% of the expected PSBI cases sought care at HPs; 74% of these cases were treated at HPs, and 90% of the cases completed their treatment  CBNC can be implemented effectively in similar contexts if it is well planned, good coordination with partners and stakeholders, uninterrupted supply is ensured, and continuous support and supervision are in place |  |

ANC: Antenatal care; CBNC: community-based newborn care; FGD: focus group discussion; HDSS: Health and Demographic Surveillance Site; HP: health post; HEW: Health Extension Worker; IDI: in-depth interview; PNC: postnatal care; PRM: performance review meeting; PSBI: possible serious bacterial infection; SYI: sick young infant; SNNP: Southern Nations, Nationalities, and Peoples’ region; WDA: Women Development Army
