# Supplementary material for: Using the Implementation Research Logic Model to design and implement community-based management of possible serious bacterial infection during COVID-19 pandemic in Ethiopia
Source: BMC Health Serv Res. 2022 Dec 13;22:1515. doi: 10.1186/s12913-022-08945-9 (PMC9745284; doi:10.1186/s12913-022-08945-9)
Supplement: Supplementary file 4 — Additional file 4. [file 12913_2022_8945_MOESM4_ESM.docx]

Additional file 4: Detailed implementation strategies for maintaining high-quality PSBI treatment when a referral is not possible amid COVID-19, June 2021

| **Challenges/Problems** | **Strategy** | **Details (what is being done)** | **Rationale/Justification** | **Outcomes targeted** | **Who is delivering** | **Who is the target** | **When is it being done** | **How often** |
| --- | --- | --- | --- | --- | --- | --- | --- | --- |
| Low confidence and competency of HEWs | Training | Provide on-the-job training in areas needing more support using existing opportunities like performance reviews | Gaps in knowledge, motivation, confidence | Fidelity, adoption, and effectiveness | Woreda health office (WorHO), and health center (HC) | HEWs | Throughout | Quarterly |
|  |  | Experience sharing meetings among well-performing HPs |  |  | HC | HEWs and HC staff | Throughout | Quarterly |
|  |  | Clinical attachment at HCs to enhance HEWs confidence on Gentamycin injection. |  |  | HC | HEWs | Throughout | Weekly |
|  | Supportive supervision | Assign trained supervisors from HCs and provide clinical mentoring on PSBI. | Gaps in knowledge, motivation, confidence | Fidelity, adoption, and effectiveness | HC | HC staff | 1^st^ Quarter | July 2021 |
|  |  | During supervision, demonstrate cases or provide case scenarios and identify knowledge gaps and needed support |  |  | Supervisors | HEWs | Throughout | Monthly |
|  | Performance review | Conduct cross-site meetings to review performance, share lessons and challenges. | Gaps in knowledge, motivation, confidence | Fidelity, adoption, and effectiveness | HC | HEWs | Throughout | Quarterly |
| Weak Health System Support | Assign a focal person for PSBI | Integrate PSBI with other child health programs at the woreda level and strengthen its implementation. | Weak Health System support | Integration, fidelity, adoption, and effectiveness enhanced | WorHO | WorHO | Throughout | Throughout |
|  |  | Work closely with PHCU to enable them to assign a focal person who follows the implementation of PSBI interventions, assign skilled supervisors to HEWs, organize and conduct PSBI performance review | Weak Health System support | A focal person assigned & fidelity, adoption, effectiveness, and feasibility | HC/WorHO | HEWs and HC | Throughout | Throughout |
|  | Strengthen supply chain | Harmonize the supply chain for drugs and equipment needed for PSBI programs. | Erratic supply and poor supply forecasting | Supply system strengthened | WorHO, HC, and HEWs | HC and HEWs | Throughout | Throughout |
|  |  | Strengthen resource mobilization, quantification, and forecasting both at HP and facility levels. |  |  | HC & HEWs | HC & HEWs | Throughout | Quarterly |
|  |  | Ensure availability of HP purchase request form and bin card, follow proper use of the formats to report, and request supplies needed. |  |  | HC | HEWs | Throughout | Throughout |
|  |  | Provide technical support during SS to address the skill gap of HEWs on how to fill and use the formats. |  |  | HC | HEWs | Throughout | Monthly |
|  |  | Identify and prioritize high caseload HFs to ensure a fair and reasonable/demand-based distribution of supply. |  |  | HC | HEWs | Throughout | Monthly |
|  |  | Establish horizontal linkage or networking of HPs to facilitate loans between adjacent HPs. |  |  | HC | HEWs | Throughout | Monthly |
|  | Advocacy Meeting | Organize and conduct advocacy meetings with woreda administrative and political authorities for budget allocation and free ambulance service arrangements for SYI. | Low attention and weak support from political authorities | Due to the attention given & PSBI supported by political authorities | WorHO | WorHO | Throughout | 1^st^ Quarter |
|  |  | Provide onsite orientation to kebele managers so they prioritize PSBI and other health-related activities. |  |  | WorHO, HC, and HEWs | Kebele managers | Throughout | 1^st^ Quarter |
|  |  | Closely work with kebele managers to make the WDA structure functional |  |  | HC & HEWs | Kebele managers & WDA | Throughout | Throughout |
|  | Establish motivation mechanism | Introduce performance-based evaluation, accountability, and motivation mechanisms for HEWs, WDAs, and PHCU staff. | No/low motivation mechanisms for HEWs and health professionals | Increased HP readiness (supplies in stock), Improved adherence to PSBI case management protocol, Increased WDA functionality | WorHO, HC  WorHO | HEWs, WDA, HC staff | Throughout | Quarterly |
|  |  | Audit and provide feedback; respect and value HPs/HEW efforts, certificate of appreciation for those who performed well, education opportunity, promotion, and reduce their abuse. |  |  | WorHO, HC | HEWs, HC staff | Throughout | Quarterly |
|  | Strengthening referral linkage | Strengthen referral and counter referral between HEWs and health facilities. Use standardized referral formats | Weak referral linkage | Increased service uptake, Improved referral linkage | HC/ WorHO | HEWs | Throughout | Throughout |
|  |  | Reduce the burden of distance and the cost of accessing referral facilities through social insurance schemes (arrange ambulance service, transportation cost reimbursement). |  |  | HC/ WorHO | HEWs | Throughout | Throughout |
|  |  | Assign referral focal person at health facility level in under five OPD. |  |  | HC | HEWs | Throughout | Throughout |
|  | Performance Review and Clinical Mentoring meeting | Strengthen and conduct regular PHCU-level integrated PRCMM (focused on PSBI intervention implementation challenges and skill gaps). HEWs should come with SYI case management recording registration when invited for PRCMM and skilled and trained health professionals should mentor them. Exchange success and challenges of PSBI/PSBI implementation and develop an action plan to mitigate challenges. | No/irregular PSBI focused PRCMM at the PHCU level | PHCUs linkage and support improved, Implementation fidelity of the implementation strategies | HC, WorHO | HC, HEWs | Throughout | Monthly |
|  | Quality improvement (QI) initiative | Introduce combined facility and community-based quality improvement initiatives. Increase reach enhancing active engagement of HEWs, WDAs/ community, and PHCU staff. Select and prioritize key facility and community-based performance indicators and worked on them. | Low quality of care provided to SYI. Low # of SYI cases properly managed at HP level. | # of sick newborns identified and managed, # of pregnant women and newborns reached with the key message, Increased service uptake | WorHO, HC, HEWs | HEWs and HC | Throughout | quarterly |
|  |  | Use RMNCH scorecard to track PSBI performance focusing on the continuum of care (timely identification of pregnant women, pregnancy care or ANC, SBA, PNC, newborn care). Use different colors to indicate progress towards the intended target based on the selected performance indicators. |  |  | WorHO, HC, HEWs | HEWs and HC | Throughout | quarterly |
|  | Integrate of activities | Work to ensure that activities are integrated at RHB, Woreda HO, PHCU, and HP levels. All departments should communicate with each other while planning activity implementation to integrate tasks that can be carried out simultaneously. | Poor integration of tasks that result in waste of resources and time | PSBI service uptake increased, Integrated service delivery, efficient use of resources | RHB, WorHO, HC | HEWs and Experts at all levels | Throughout | Throughout |
| Sub-optimal community engagement | Awareness creation | Use any existing opportunities for awareness creation regarding available child health services at the HP level (community meetings, school, home visits, religious institutions, etc.). | Community misunderstandings about newborn illnesses and low health-seeking behavior for SYI | Increased community awareness, Improved health-seeking behavior for SYI. | HEWs, WDA, and kebele managers | Community | Throughout | Throughout |
|  |  | Provide counseling services on the pathways to SYI care and treatment (at service delivery points). |  |  | HEWs, HC staff | Community | Throughout | Throughout |
|  |  | Engage and use influential individuals within the community for awareness creation and maintain community trust in PSBI services (religious leaders, community influential, etc.). |  |  | HEWs, supervisors, kebele managers | Community | Throughout | Throughout |
|  | HP exhibition/ open HP 8 hrs. per day | Make HPs open 8 hours/day by assigning an adequate number of HEWs per kebele (minimum 2 HEWs per kebele) | Irregular opening of HP | Improved access to services, Increased service uptake | HC, WorHO, HEWs, kebele managers | HEWs | Throughout | Daily |
|  |  | Minimize assignment of non-health-related roles to HEWs and integrate activities, allocate allowance for weekend-based work. |  |  | HC, kebele manager | HEWs | Throughout | Throughout |
|  | Kebele-level multi-sectoral meeting | Provide technical support to HEWs to closely work with kebele managers to facilitate regular kebele-level multi-sectoral meetings. | HEW workload, assignment of non-health-related roles to HEWs. | Reduced workload for HEWs/ minimized assignment of non-health-related roles to HEWs | HC, Kebele admin. | HEWs and kebele managers | Throughout | Throughout |

ANC: antenatal care; HC: health center; HP: health post; HEW: Health Extension Worker; OPD: outpatient department; RHB: regional health bureau; SBA: skilled delivery; SS: supportive supervision; PHCU: Primary Health Care Unit; PNC: postnatal care; PRCMM: performance review and clinical mentoring meeting; PSBI: possible serious bacterial infection; WDA: Women Development Army; WorHO: Woreda health office
